# Efficacy and immunogenicity of different BCG doses in BALB/c and CB6F1 mice when challenged with H37Rv or Beijing HN878

**DOI:** 10.1038/s41598-021-02442-5

**Published:** 2021-12-02

**Authors:** Bhagwati Khatri, James Keeble, Belinda Dagg, Daryan A. Kaveh, Philip J. Hogarth, Mei Mei Ho

**Affiliations:** 1 Bacteriology Division, National Institute for Biological Standards and Control, South Mimms, Potters Bar, Hertfordshire, EN6 3QG UK; 2 Department of Bacteriology, Animal and Plant Health Agency, Addlestone, Surrey KT15 3NB UK

**Keywords:** Cell biology, Developmental biology, Immunology, Microbiology, Diseases

## Abstract

Two strains of mice (BALB/c and CB6F1) were vaccinated with a range of Bacille Calmette-Guérin (BCG) Danish doses from 3 × 10^5^ to 30 CFU/mouse, followed by aerosol infection with *Mtb* (H37Rv or West-Beijing HN878 strain). The results indicated that both strains of mice when infected with HN878 exhibited significant protection in their lungs with BCG doses at 3 × 10^5^—3000 CFU (BALB/c) and 3 × 10^5^—300 CFU (CB6F1). Whereas, a significant protection was seen in both strains of mice with BCG doses at 3 × 10^5^—300 CFU when infected with H37Rv. A significant increase in the frequencies of BCG-specific IFNγ^+^ IL2^+^ TNFα^+^ CD4 T cells in the BCG doses at 3 × 10^5^—3000 CFU (BALB/c) and 3 × 10^5^—300 CFU (CB6F1) was seen. The IFNγ^+^ IL2^+^ TNFα^+^ CD4 T cells correlated with the *Mtb* burden in the lungs of HN878 infected mice (BALB/c and CB6F1) whereas, IFNγ^+^ TNFα^+^ CD4 T cells correlated with the BALB/c mice infected with H37Rv or HN878. The BCG dose at 3000 CFU (an equivalent single human dose in the mice by body weight) is protective in both strains of mice infected with H37Rv or HN878 and may serve an interesting dose to test new TB vaccine in a preclinical animal model.

## Introduction

Bacille Calmette-Guérin (BCG) is one of the most widely used injectable vaccines, administered predominantly in neonates, but confers incomplete and variable protection against pulmonary tuberculosis (TB) in humans^1–3^. Despite its widespread use, an estimated 1.4 million people in 2019 died due to this disease^4^. Studies in the field of infectious diseases, especially for intracellular pathogens, acknowledges the complex interplay of the magnitude, speed and qualitative nature of protective immune responses by chronic infections (such as TB, leprosy, leishmaniasis) which is not as straightforward as an effective antibody response against extracellular pathogens^5^.

Although there are many animal models used in TB research for testing vaccine candidates or drugs against TB. Having recognised the limitations of animal models in terms of predicting outcomes in humans, moving forward, it is vital to improve animal models wherever possible^6,7^. Among all animal models, the mouse model is the most preferred choice, mainly due to the small animal size, vast array of commercially available reagents and the low cost of running an experiment. From the decades of published TB challenge studies^7–10^, it is evident to state that BCG protective responses against TB in mice vary by the choice of mouse strain^11^, strains of *Mycobacteria tuberculosis* (*Mtb*) for infection, route of immunisation and the interval between BCG vaccination and *Mtb* challenge. Our aim in this study is to optimise and analyse some of these parameters such as the choice of mouse strain and the efficacy of different doses of BCG against two strains of *Mtb* namely, laboratory TB strain H37Rv and a virulent clinical strain, W-Beijing HN878 (HN878). HN878 have shown to reduce TH1 responses and associated with decreased survival rate in mice infected with HN878 when compared to H37Rv^12^.

In this study, each strain of *Mtb* will be used in a head-to-head comparison using two types of mouse strains, BALB/c and CB6F1 (a cross between female BALB/c and male C57BL/6). The CB6F1 mouse strain have an increased diversity of major histocompatibility complex class II genes (I-A^b^, I-A^d^, I-E^d^) compared to the I-E^d^ in BALB/c and I-A^b^ in C57BL/6 mice thereby presenting antigens to CD4^+^ T cells which triggers coordination and regulation of effector cells^13^. The rationale for conducting this study is for dose sparing of BCG vaccine, refinement of the TB mouse model and evaluating protective responses of lower BCG dose in the mouse which is equivalent to single human dose per weight of the human infant.

The study was divided into two parts; 1. H37Rv or HN878 challenge—different doses of BCG were evaluated by measuring *Mtb* burden in the lungs and spleen of mice infected with either of the *Mtb* strain and; 2. Immunogenicity evaluation—cellular immune responses were analysed using flow cytometry, IFN-γ ELISPOT and multiplex cytokine/chemokine assays in BCG vaccinated and control mice. The overall results demonstrated differences in the protection and cellular immune responses in both strains of mice. Our findings will benefit refinement of the TB challenge mouse model for the preclinical testing and selection of new TB vaccines.

## Results

**Figure 1:**
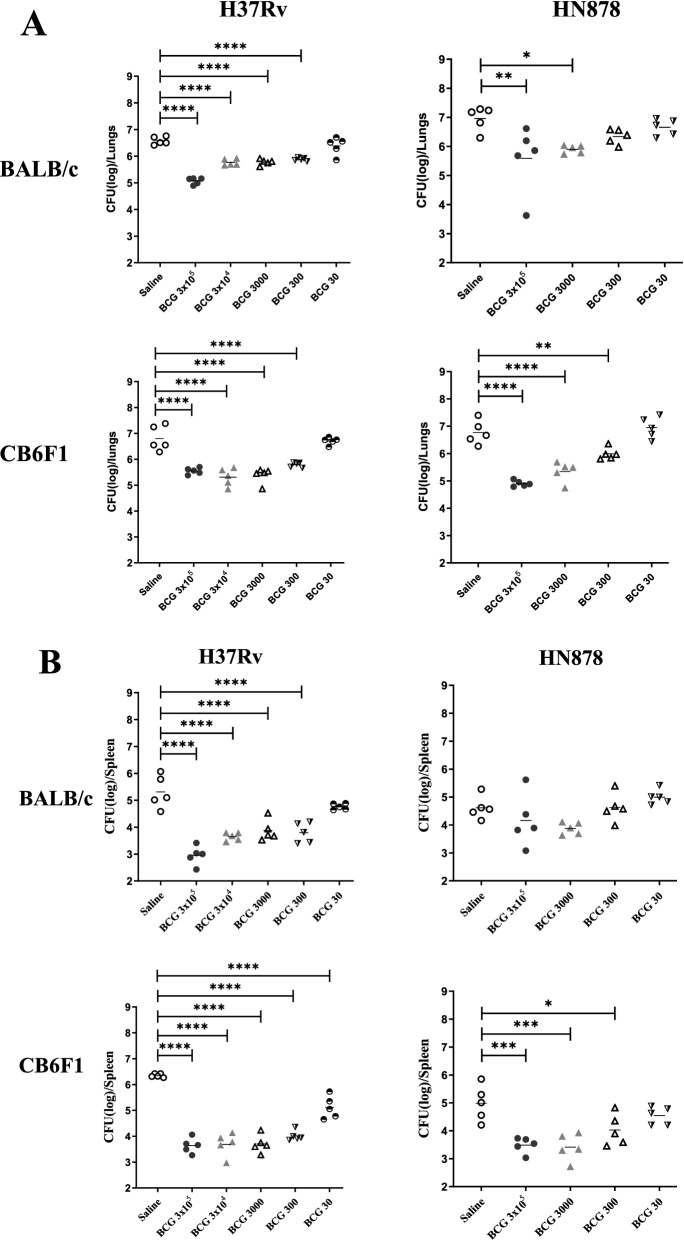
The CFU data for mice (five mice/group)—BALB/c and CB6F1, vaccinated with different BCG doses and challenged with HN878 and H37Rv. Four weeks post infection, the lungs and spleen were harvested, homogenates were plated on 7H11 agar plates and bacterial loads were determined as CFU/ml. Data is representative of two independent experiments for H37Rv challenge and one experiment for HN878 challenge. (**A**) Lungs CFU data as Log_10_ protection for the vaccinated BALB/c and CB6F1 mice infected with H37Rv or HN878. (**B**) Spleen CFU data as Log_10_ protection for the vaccinated BALB/c and CB6F1 mice infected with H37Rv or HN878. One-way ANOVA, Tukey’s multiple comparison test was performed for BALB/c and CB6F1 mice infected with H37Rv and HN878 strains. *ρ < 0.05, **ρ < 0.005, ***ρ < 0.0005, ****ρ < 0.0001.

In the initial experiment where BALB/c and CB6F1 mice were vaccinated with a full range of BCG doses (3 × 10^5^, 3 × 10^4^, 3000, 300 or 30 CFU/mouse) followed by H37Rv challenge, a comparable protection was seen in the lungs of mice vaccinated with the BCG doses at 3 × 10^4^ and 3000 CFU (Fig. 1A). Considering the principle of replacement, reduction and refinement (3Rs) in the experimental design using animals, BCG dose at 3 × 10^4^ CFU was omitted from HN878 challenge experiments and the immunogenicity evaluation study. Concentrations of all serially diluted BCG doses (CFU/ml) were plated in duplicate onto 7H11 agar plates and confirmed to be within the accepted range (Supplementary Table 1). The concentrations of an infection inocula for H37Rv or HN878 were also confirmed for each challenge experiment and the representative average values were 3.6 + 0.85 (+SD) × 10^6^ CFU/ml or 4 + 0.91 (+SD) × 10^6^ CFU/ml respectively (Supplementary Table 2). Both infection challenges were used to obtain low dose aerosol infection of approximately 100 CFU per mouse. BALB/c and CB6F1 mice were infected together with either H37Rv or HN878 in all the representative challenge experiments. To check the uptake of low dose infection of 100 CFU/mouse for H37Rv and HN878, the lungs of 1 day post infected BALB/c mice were harvested and CFU/ml determination were confirmed. Supplementary Table 2 shows the uptake result of H37Rv—average 71 + 63 (+SD) CFU and HN878—average 144 + 64 (+SD) CFU per mouse. Due to the logistics of working in the containment level III, immunogenicity evaluation was not performed in mice post infection with H37Rv or HN878.

### BCG protection in BALB/c and CB6F1 mice varies when challenged with H37Rv or HN878

Four weeks after BCG vaccination, BALB/c and CB6F1 mice were infected with either H37Rv or HN878. Animals were sacrificed at 4 weeks post infection and the lungs and spleen were harvested to determine *Mtb* burden. Figure 1A shows the graphical representation of the protection data expressed as Log_10_ CFU in the lungs of BALB/c and CB6F1 mice infected with either H37Rv or HN878. Tables 1A,B represent the differences in the mean of Log_10_ CFU/organ between all the BCG vaccinated and the control groups, including within BCG vaccinated groups in the lungs and spleen of both strains of mice when infected with H37Rv or HN878. For the lowest BCG dose at 30 CFU, *Mtb* burden (Log_10_ CFU/organ) in the lungs of both mouse strains infected with either H37Rv or HN878 were equivalent to the control group (Fig. 1A; Table 1A,B).

**Table 1:** Lung and spleen protection data for BALB/c and CB6F1 mice when infected with H37Rv or HN878 strains. The values in the Log_10_ protection data is the difference in the means of control group when compared to the BCG dose groups and the difference within BCG groups. Data is representative of two independent experiments for H37Rv and one experiment for HN878. Values representing difference for the mean Log_10_ of the BCG and control groups (left column)—mean Log_10_ of the BCG groups (Top Row). A, Difference between the means of the lungs and spleen of Log_10_ values for BALB/c and CB6F1 mice infected with H37Rv. B, Difference between the means of the lungs and spleen of Log_10_ values for BALB/c and CB6F1 mice infected with HN878. One-way ANOVA, Tukey’s multiple comparison test was performed for BALB/c and CB6F1 mice infected with H37Rv or HN878. *ρ < 0.05, **ρ < 0.005, ***ρ < 0.0005, ****ρ < 0.0001.

A												
Groups	BALB/c—Lungs (Log_10_)						CB6F1—Lungs (Log_10_)					
	Control	3 × 10^5^	3 × 10^4^	3000	300	30	Control	3 × 10^5^	3 × 10^4^	3000	300	30
Control	N/A	1.51****	0.81****	0.80****	0.70****	0.2	N/A	1.26****	1.50****	1.42****	1.01***	0.1
3 × 10^5^	– 1.51****	N/A	– 0.70****	– 0.71****	– 0.81****	– 1.32****	– 1.26****	N/A	0.24	0.16	– 0.24	– 1.16****
3 × 10^4^	– 0.81****	0.70****	N/A	– 0.01	– 0.12	– 0.62****	– 1.50****	– 0.24	N/A	– 0.07	– 0.48	– 1.36****
3000	– 0.80****	0.71****	0.01	N/A	– 0.11	– 0.61***	– 1.42****	– 0.16	0.07	N/A	– 0.41	– 1.32****
300	– 0.70****	0.81****	0.12	0.11	N/A	– 0.50**	– 1.01***	0.24	0.48	0.41	N/A	– 0.91***
30	– 0.2	1.32****	0.62****	0.61***	0.50**	N/A	– 0.1	1.16****	1.36****	1.32****	0.91***	N/A

Groups	BALB/c—Spleen (Log_10_)						CB6F1—Spleen (Log_10_)					
	Control	3 × 10^5^	3 × 10^4^	3000	300	30	Control	3 × 10^5^	3 × 10^4^	3000	300	30
Control	*N/A*	2.36****	1.66****	1.44****	1.51****	0.54	*N/A*	2.71****	2.66****	2.65****	2.33****	1.23****
3 × 10^5^	– 2.36****	*N/A*	– 0.69	– 0.92**	– 0.85*	– 1.81****	– 2.71****	*N/A*	– 0.05	– 0.06	– 0.38	– 1.48****
3 × 10^4^	– 1.66****	– 0.69	*N/A*	– 0.23	– 0.15	– 1.12***	– 2.66****	0.05	*N/A*	– 0.01	– 0.33	– 1.43****
3000	– 1.44****	0.92**	0.23	*N/A*	0.08	– 0.89**	– 2.65****	0.06	0.01	*N/A*	0.31	– 1.41****
300	– 1.51****	0.85*	0.15	– 0.08	*N/A*	– 0.97**	– 2.33****	0.38	0.33	– 0.31	*N/A*	– 1.10***
30	– 0.54	1.81****	1.12***	0.89**	0.97**	*N/A*	– 1.23****	1.48****	1.43****	1.41****	1.10***	*N/A*

B												
Groups	BALB/c—Lungs (Log_10_)						CB6F1—Lungs (Log_10_)					
	Control	3 × 10^5^	3 × 10^4^	3000	300	30	Control	3 × 10^5^	3 × 10^4^	3000	300	30
Control		1.37**		1.06*	0.62	0.3		1.87****		1.42****	0.78**	– 0.81
3 × 10^5^	– 1.37**	*N/A*		– 0.31	– 0.75	– 1.07	– 1.87****	*N/A*		– 0.44	– 1.086***	– 2.05****
3 × 10^4^												
3000	– 1.06*	0.31		*N/A*	– 0.44	– 0.76	– 1.42****	0.44		*N/A*	– 0.65*	– 1.61****
300	– 0.62	0.75		0.44	*N/A*	– 0.32	– 0.78**	1.086***		0.65*	*N/A*	– 0.96**
30	– 0.3	1.07		0.76	0.32		0.81	2.05****		1.61****	0.96**	*N/A*

Groups	BALB/c—Spleen (Log_10_)						CB6F1—Spleen (Log_10_)					
	Control	3 × 10^5^	3 × 10^4^	3000	300	30	Control	3 × 10^5^	3 × 10^4^	3000	300	30
Control	*N/A*	0.46	Not done	0.74	– 0.01	– 0.38	*N/A*	1.5***		1.57***	0.96*	0.44
3 × 10^5^	– 0.46	*N/A*		0.29	– 0.47	– 0.84	– 1.5***	*N/A*		0.07	– 0.54	– 1.06*
3 × 10^4^								*N/A*		*N/A*		
3000	– 0.74	– 0.29		*N/A*	– 0.76	– 1.12	– 1.57***	0.07		*N/A*	– 0.61	– 1.13**
300	0.01	0.47		0.76	*N/A*	– 0.37	– 0.96*	0.54		0.61	*N/A*	– 0.52
30	0.38	0.84		1.12	0.37	*N/A*	– 0.44	1.06*		1.13**	0.52	*N/A*

H37Rv infected BALB/c and CB6F1 mice vaccinated with BCG at 3 × 10^5^—300 CFU, displayed significant protection (Fig. 1A and Table 1A,B) in the lungs when compared to the control group. The protection between the BCG groups for BALB/c and CB6F1 mice differed. BALB/c mice infected with H37Rv showed significantly more protection in the lungs of the highest BCG dose 3 × 10^5^ CFU (Table 1A) when compared to all the lower doses of BCG at 3 × 10^4^, 3000, 300 and 30 (Table 1A). In comparison to BALB/c, H37Rv infected CB6F1 mice displayed comparable protection in the lungs with the highest dose of BCG 3 × 10^5^ CFU when compared to all the lower doses of BCG at 3 × 10^4^, 3000, 300, except 30 (Table 1A). In the spleen (Fig. 1B), both strains of mice showed significantly more protection in the groups vaccinated with BCG at 3 × 10^5^, 3 × 10^4^, 3000 and 300 CFU when compared to the control group. In addition, spleen of H37Rv infected CB6F1, but not BALB/c mice, BCG dose at 30 CFU showed significant protection when compared to the control group, Fig. 1B and Table 1A.

When infected with HN878, BALB/c mice only demonstrated significant protection in the lungs with BCG doses at 3 × 10^5^ and 3000 CFU, while CB6F1 mice showed significant protection in the lungs with a BCG dose as low as 300 CFU when compared to the control group (Fig. 1A and Table 1B). In addition, CB6F1 mice displayed a dose response pattern in the protection showing the difference of Log_10_ CFU/lungs of different BCG groups compared to the control group: 1.87 (BCG 3 × 10^5^), 1.42 (BCG 3000) and 0.78 (BCG 300) when compared to the control group (Fig. 1A, Table 1B). In the spleen (Fig. 1B and Table 1B), CB6F1 mice showed significant protection with BCG at 3 × 10^5^, 3000 and 300 CFU whereas, BALB/c mice failed to exhibit protection for all the BCG vaccinated groups when compared to the control group.

### Multifunctional CD4 T cells producing IFNγ, IL2 and TNFα

**Figure 2:**
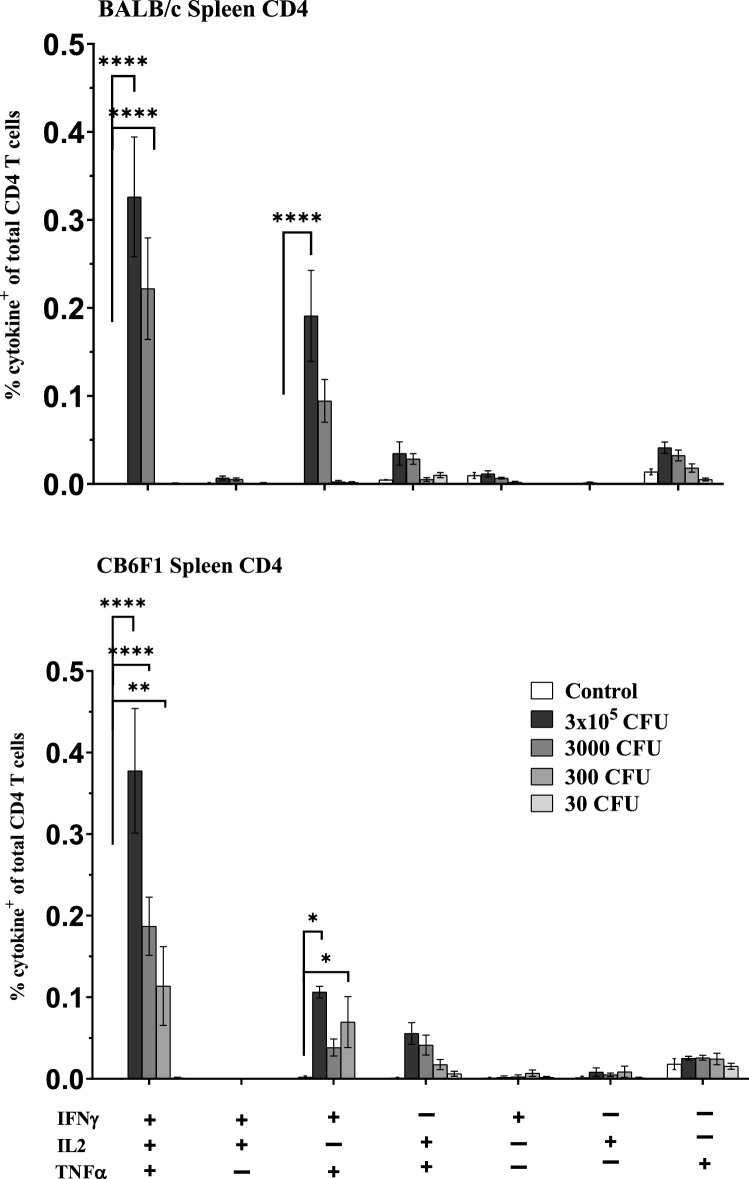
Vaccination induced resident multifunctional CD4^+^ cells in the spleen of BALB/c and CB6F1 mice. Using flow cytometry, the frequency of multifunctional CD4^+^ CD44^hi^ CD62L^lo^ cells at 6 weeks following BCG immunization. A, splenocytes from BALB/c and CB6F1 mice (five mice/group) from the control and all BCG groups were isolated, stimulated with M7 protein cocktail and stained by ICS. Data is the representative of one independent experiment for both strains of mice. Bars represent mean (±SE) percentage frequency of cells of indicated T cell phenotype as a percentage of total CD4+ cells. Two-way ANOVA, Tukey’s multiple comparison was performed, *ρ < 0.05, **ρ < 0.005, ***ρ < 0.0005, ****ρ < 0.0001.

In order to better resolve the functionality of the responding T cells induced post BCG vaccination, M7 stimulated splenocytes from the groups (control, BCG at 3 × 10^5^, 3000, 300 and 30 CFU) of BALB/c and CB6F1 mice were subsequently interrogated by intracellular staining (ICS) using 11 colour flow cytometric analysis. The frequency of CD4 T cells producing any combination of IFNγ, IL2, TNFα and IL17a production was assessed. As shown in Fig. 2, BALB/c and CB6F1 mice induced significantly more multifunctional IFNγ^+^IL2^+^TNFα^+^ CD4 T cells with BCG at 3 × 10^5^–3000 CFU and 3 × 10^5^–300 CFU, respectively when compared to the control group. Within the BCG vaccinated groups, 3 × 10^5^ CFU induced a significantly higher frequency of multifunctional IFNγ^+^IL2^+^TNFα^+^ CD4 T cells in BALB/c when compared to 3000 CFU and in CB6F1 mice when compared to 3000 and 300 CFU. The frequency of CD4 T cells expressing IL17a in the BCG vaccinated groups were low (Supplementary Fig. 1).

For both strains of mice, cytokine producing splenocytes IFNγ^+^ TNFα^+^ CD4 T cells were significantly induced in BCG at 3** × **10^5^–3000 CFU when compared to the control group (Fig. 2). In addition, CB6F1 mice induced IFNγ^+^ TNFα^+^ CD4 T cells in 300 CFU compared to the control group (Fig. 2). BCG at 30 CFU group failed to induce multifunctional IFNγ^+^IL2^+^TNFα^+^ or IFNγ^+^ TNFα^+^ CD4 T cells in both strains of mice. The cytokine producing CD4 T cells detected here, displayed a CD44^hi^CD62L^lo^ phenotype, indicative of CD4 T Effector Memory (TEM), data not shown (gating strategy in Supplementary Fig. 2).

To understand the significance of correlation between the means of BCG-specific multifunctional CD4 TEM cells in the spleen and *Mtb* burden in the lungs of H37Rv or HN878 infected BALB/c and CB6F1 mice in the equivalent vaccine groups, we performed correlation coefficient analysis. As shown in Table 2, the correlations were analysed between the mean of IFNγ^+^IL2^+^TNFα^+^, IFNγ^+^ TNFα^+^, IFNγ^+^IL2^+^, IL2^+^TNFα^+^ CD4 T cells and the mean of *Mtb* burden as expressed in Log_10_ CFU/lungs. Multifunctional IFNγ^+^IL2^+^TNFα^+^ CD4 T cells were significantly inversely correlated with HN878 infected BALB/c (r =  − 0.918, ρ = 0.0278) and CB6F1 mice (r =  − 0.958, ρ = 0.0103). However, correlation was not seen with H37Rv infected BALB/c and CB6F1 mice. For IFNγ^+^ TNFα^+^ CD4 T cells, a significantly inverse correlation was seen in the BALB/c mice infected with either H37Rv (r =  − 0.9038, ρ = 0.0353) or HN878 (r =  − 0.9043, ρ = 0.035). Again, for IFNγ^+^ TNFα^+^ CD4 T cells, correlation was not seen in CB6F1 mice infected with H37Rv or HN878. For IL2^+^ TNFα^+^ CD4 T cells, a significantly inverse correlation was observed in the HN878 infected BALB/c (r =  − 0.9094, ρ = 0.0323), CB6F1 (r =  − 0.9739, ρ = 0.005) and H37Rv infected CB6F1 mice (r =  − 0.8816, ρ = 0.048).

**Table 2:** Correlation of BALB/c lungs (Log_10_ CFU/ml) and CB6F1 lungs (Log_10_ CFU/ml) mice vaccinated and challenged with H37Rv or HN878 versus multifunctional CD4 T cells of M7 stimulated splenocytes from mice vaccinated with BCG. Correlation analysis. Correlation analysis between the TB burden in the lungs (Log_10_ CFU/ml) of BALB/c and CB6F1 mice infected with H37Rv or HN878 versus correlation analysis between the multifunctional CD4 T cells. Correlation coefficient was assessed using Pearson’s two-tailed correlation test with ρ value (*ρ < 0.05, **ρ < 0.005).

	BALB/c H37Rv lungs (Log_10_ CFU/ml)	CB6F1 H37Rv lungs (Log_10_ CFU/ml)	BALB/c HN878 lungs (Log_10_ CFU/ml)	CB6F1 HN878 lungs (Log_10_ CFU/ml)
CD4 T cells expressing cytokines	*P* value (Pearson r)	*P* value (Pearson r)	*P* value (Pearson r)	*P* value (Pearson r)
IFNγ^+^IL2^+^TNFα^+^	0.052 (− 0.875)	0.0899 (− 0.8189)	*0.0278 (− 0.918)	*0.0103 (− 0.958)
IFNγ^+^IL2^+^	0.0943 (− 0.8129)	N/A	0.0515 (− 0.8759)	N/A
IFNγ^+^TNFα^+^	*0.035 (− 0.9038)	0.1162 (− 0.7844)	*0.0353 (− 0.9043)	0.0589 (− 0.8641)
IL2^+^TNFα^+^	0.0802 (− 0.8325)	*0.048 (− 0.8816)	*0.0323 (− 0.9094)	**0.005 (− 0.9739)

These results highlight the induction of multifunctional IFNγ^+^IL2^+^TNFα^+^, IFNγ^+^ TNFα^+^ CD4 T cells and IL2^+^ TNFα^+^ CD4 T cells in both strains of mice exhibiting dose response to the vaccine. The highest BCG dose group, 3 × 10^5^ CFU induced significantly higher frequency of CD4 TEM cells when compared to the BCG at 3000—30 CFU groups. In addition, the IFNγ^+^IL2^+^TNFα^+^ CD4 T cells inversely correlated with the HN878 infected BALB/c and CB6F1 mice which demonstrates: the higher the proportion of these cells, the lower is *Mtb* burden in the lungs of subsequently infected mice. Interestingly, IFNγ^+^ TNFα^+^ CD4 T cells inversely correlated with the *Mtb* burden in the BALB/c mice with both *Mtb* challenge strains, but not with CB6F1 mice.

### Differential expression of cytokines/chemokines in BALB/c and CB6F1 mice

**Figure 3:**
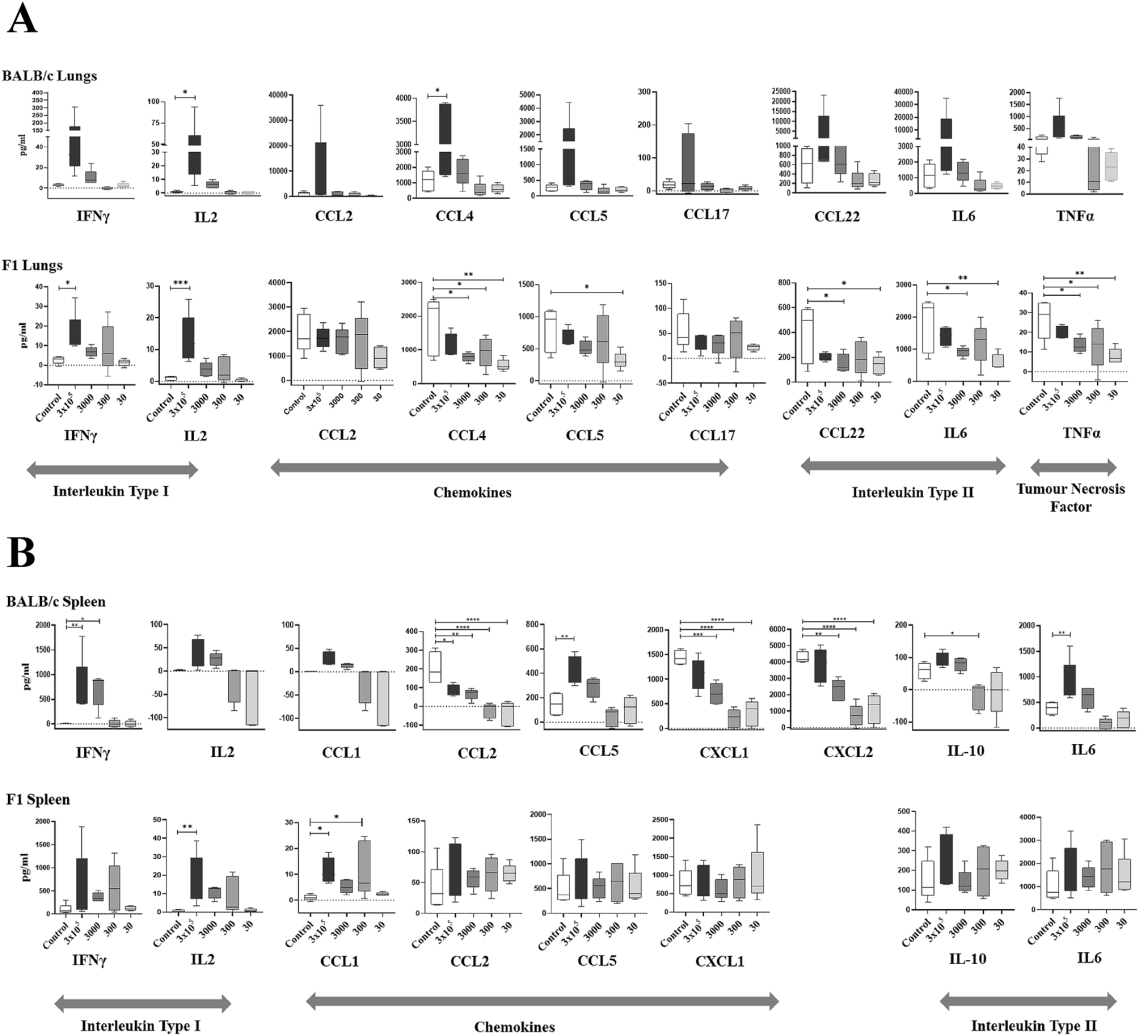
Cytokines/chemokines expression in the M7 stimulated the lung cells and splenocytes from BALB/c and CB6F1 mice vaccinated with various BCG doses. Data is the representative of one independent experiment for both strains of mice. Concentration of cytokines and chemokines were obtained using Luminex xMAP^®^ Technology. The concentration of each cytokine or chemokine (pg/ml) was obtained by subtracting concentration of M7-stimulated with the no antigen cells and used as data for graphs (**A** and **B**). A representative graph of cytokines/chemokines expressed in pg/ml after 3 days of stimulated lung cells (top graphs) splenocytes (bottom graphs) of BALB/c and CB6F1 mice for all groups. Bars representing mean (± S.E.) pg/ml. One-way ANOVA, Dunnett’s multiple comparison test, *ρ < 0.05, **ρ < 0.005, ***ρ < 0.0005 was performed for (**A** and **B**).

The selection of cytokines/chemokines were mainly based on the TH1, TH2 responses and consideration was given in choosing overlapping cytokines/chemokines which were induced after BCG vaccination and TB infection. Amonst these overlapping cytokines/chemokines are pro-inflammatory cytokines; IFNγ, TNFα, IL2, IL1β and IL16; TH2 cytokines: IL4, IL6, the regulatory cytokine IL-10; chemokines (IP-10, MIP-1α and IL-8) and growth factor (GM-CSF). The lung cells and splenocytes, isolated from the BCG vaccinated and control groups were stimulated with M7 protein cocktail for 3 days after which they were collected. Analyses of cytokines and chemokines, as listed in the Table 3, were performed in the supernatants of cultured cells (stimulated and unstimulated) using Bio-Plex Pro Mouse Chemokine Panel. Figure 3A,B are the representative graphs of cytokines/chemokines obtained from the lungs or spleen of BALB/c or CB6F1 mice and the Supplementary Table 3 tabulates all concentrations of cytokines/chemokines from the lungs and spleen of BALB/c and CB6F1 mice. Groups vaccinated with BCG at 3 × 10^5^ CFU exhibited greater variability in the expression of cytokines/chemokines in the lungs of BALB/c mice compared to the other BCG vaccinated groups. For the ease of result presentation, significance in all the BCG vaccinated groups have been compared with the control group, unless depicted otherwise.

**Table 3:** Classification of examined cytokines and chemokines in the M7 stimulated supernatants of splenocytes and lung cells from BCG vaccinated BALB/c and CB6F1 mice.

Chemokine	CCL	CTACK/CCL27, Eotaxin/CCL11, Eotaxin-2/CCL24, I-309/CCL1, MCP-1/CCL2, MCP-2/CCL8*, MCP-3/CCL7, MCP-5/CCL12, MDC/CCL22, MIP-1α/CCL3, MIP-1β/CCL4, MIP-3α/CCL20, MIP-3β/CCL19, RANTES/CCL5, MCP-3/CCL7 and TECK/CCL25*
	CXCL	BCA-1/CXCL13, Fractalkine/CX3CL1, ENA-78/CXCL5, I-TAC/CXCL11, KC/CXCL1, MIP-2/CXCL2*, SCYB16/CXCL16, SDF-1α/CXCL12, IP10/CXCL10
TNF		TNFα
Interleukins Type I	Th1 cytokines growth factors and Interferons	IFNγ, IL2, IL16, GM-CSF, IL1β
Interleukins Type II	Interleukins	IL4, IL10, IL6

*Not analysed for CB6F1 mice (Splenocytes and lung cells) as absent in the Bio-Plex Pro Mouse Chemokine Panel.

As expected, secreted IFNγ concentration in the splenocytes of BALB/c mice (Fig. 3B) were detectable at significantly higher concentration in the BCG at 3 × 10^5^ and 3000 CFU groups. However, although IFNγ responses were not significantly different, the splenocytes of CB6F1 mice displayed an elevated response in the BCG at 3 × 10^5^—300 CFU groups (Fig. 3B). Interestingly, a significantly higher IL2 concentration (Fig. 3A,B) in the BCG at 3 × 10^5^ CFU group for the CB6F1 (lungs and spleen) and BALB/c (lungs) mice were observed. The elevated responses of IL2 were also detected in the lung cells and splenocytes of CB6F1 (BCG at 3000—300 CFU groups) and BALB/c (BCG at 3000 CFU group) mice.

Surprisingly, the concentrations of secreted chemokines, CCL2, CXCL1 and CXCL2 were significantly lower from the splenocytes of all the BCG vaccinated compared to the control group for BALB/c, but not CB6F1 mice. For CB6F1 mice, secreted IL6, TNFα, CCL4 and CCL22 concentrations from the lung cells (Fig. 3A) were lower in all the BCG vaccinated groups and displaying significant differences in the BCG at 3000—30 CFU groups when compared to the control group. In contrast to the CB6F1 mice, the lung cells of BALB/c mice secreted significantly higher concentrations of IL6 and CCL4 for 3 × 10^5^ CFU BCG group when compared to the control group. The rest of the cytokines/chemokines shown in Fig. 3A,B, secreted elevated concentrations in the 3 × 10^5^ CFU BCG group when compared to the control and 3000—30 CFU BCG groups.

### Correlation of cytokine/chemokine with TB burden in the lungs of BALB/c and CB6F1 mice

Assessing the magnitude and frequency of IFNγ has remained the standard measurement of vaccine induced responses. We sought to investigate the correlation of vaccine induced IFNγ and other cytokines/chemokines with the *Mtb* burden in the lungs of BALB/c and CB6F1 mice subsequently infected with either H37Rv or HN878.

Firstly, correlation between the IFNγ concentration and other cytokine/chemokine concentrations from the stimulated cell supernatants were investigated (Supplementary Table 4). For the correlation analysis, the mean of IFNγ concentration was correlated with the mean concentration of other cytokines/chemokines from the supernatants of splenocytes/lung cells of all BCG doses for the BALB/c and CB6F1 mice. For example, the mean of IFNγ concentration from BALB/c splenocytes was correlated with the mean concentrations of other cytokines/chemokines within the same supernatant sample. As shown in the Supplementary Table 4, IFNγ responses in the splenocytes of BALB/c mice significantly correlated with IL6 and CCL5. In the splenocytes of CB6F1 mice, IL2, CCL1, CCL5 and GM-CSF were significantly correlated with the IFNγ responses. In addition, the lungs of BALB/c mice displayed correlations of IFNγ with many cytokines which did not show correlation in CB6F1 mice such as, CXCL5, GM-CSF, IL1β, IL6, IL16, CXCL11, CCL2, CCL7, CCL22, CCL5, CXCL16, CCL17 and TNFα. The cytokines that displayed significant correlation with the IFNγ in the lungs of both strains of mice were CCL1, IL2 and IP10.

To gain further insight for whether the cytokines/chemokines which correlated with the IFNγ responses in the BCG vaccinated groups (Supplementary Table 4) also correlated with the *Mtb* burden in the lungs of BALB/c or CB6F1 mice. Supplementary Table 5 shows the correlation between all cytokine concentrations (pg/ml) and *Mtb* burden in the lungs of BALB/c and CB6F1 mice infected with H37Rv or HN878. It was surprising from the data that none of the cytokines from the lungs of BALB/c mice which were correlated with IFNγ response from the Supplementary Table 4, displayed any correlation with the *Mtb* burden in the lungs of BALB/c mice infected with either H37Rv or HN878 (except significantly inverse correlation of IFNγ in HN878 infected BALB/c mice). However, for the CB6F1 mice, CXCL13, GM-CSF and IL2 showed significantly inverse correlation with the *Mtb* burden in the lungs of CB6F1 mice infected with the both *Mtb* strains, H37Rv or HN878.

### IFNγ secreting cells by ELISPOT assay

**Figure 4:**
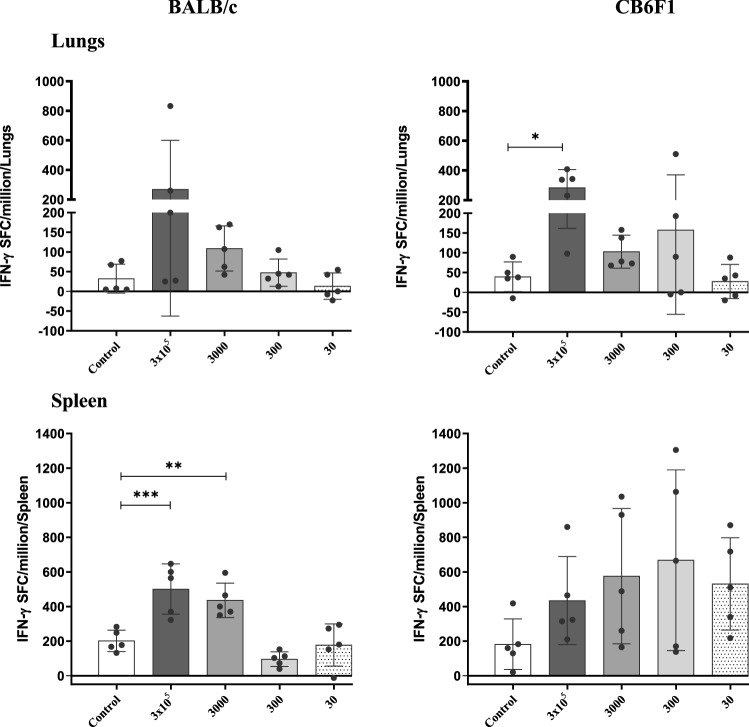
IFNγ secreting cells in the lungs and spleen of BALB/c and CB6F1 mice. The lung cells and splenocytes were isolated from 6 weeks post BCG vaccinated BALB/c and CB6F1 mice (five mice/group) and the frequency of IFNγ secreting cells evaluated by ex vivo ELISPOT. Data is the representative of two independent experiments for BALB/c and one experiment for CB6F1 mice. Approximately 2 × 10^5^ cells were cultured with or without M7 protein cocktail and developed by ELISPOT assay. For the data interpretation, the frequency of M7-stimulated IFNγ secreting cells were subtracted from the frequency of IFNγ secreting cells with no antigen stimulation. Bars representing mean (±S.E.) Spot Forming Cells (SFC) per million. Statistical analysis: One-way ANOVA, Dunnett’s multiple comparison test was performed. *ρ < 0.05, **ρ < 0.005, ***ρ < 0.0005.

Given the established critical role of IFNγ in controlling TB infection^14,15^, the frequency of antigen-specific IFNγ secreting cells were evaluated ex vivo by ELISPOT from the lungs and spleen of BCG vaccinated mice. All lung cells and splenocytes were stimulated with or without a defined M7 protein cocktail. For data interpretation, the frequency of M7-stimulated IFNγ secreting cells were subtracted from the frequency of IFNγ secreting cells with no antigen stimulation. In the lungs of CB6F1 mice (Fig. 4), only BCG at 3 × 10^5^ CFU found to have significantly higher frequency of IFNγ secreting cells when compared to the control group, whereas BCG at 3000 and 300 CFU displayed higher responses compared to the control group. In the spleen of BALB/c mice (Fig. 4), significantly higher frequency of BCG-specific IFNγ secreting cells was evident in the mice vaccinated with BCG at 3 × 10^5^ and 3000 CFU when compared to the control group, whereas they were equivalent to the control group in the BCG at 300 and 30 CFU groups. In the spleen of CB6F1 mice, albeit the frequency of IFNγ secreting cells were similar in all the BCG vaccinated groups, BCG at 3 × 10^5^—30 CFU displayed higher frequency of these cells when compared to the control group.

Together, these results highlight that in the lungs and spleen of BALB/c and CB6F1 mice, higher frequency of BCG-specific IFNγ secreting cells were only detected in the groups that received either BCG at 3 × 10^5^ or 3000 and not the lower doses.

To understand whether the ex vivo IFNγ secreting cells correlate with the *Mtb* burden in the lungs of BALB/c and CB6F1 mice infected with H37Rv or HN878, a correlation analysis was performed (Supplementary Table 6). Significantly inverse correlation of IFNγ secreting cells was only observed in the lungs of BALB/c mice infected with H37Rv.

## Discussion

NIBSC have been supporting the international efforts to develop new TB vaccines using a specialised murine aerosol TB challenge model for testing new vaccine candidates. As a recognised WHO reference laboratory, NIBSC has established the WHO reference reagents for BCG vaccine of various substrains^16,17^.Our laboratory has been involved in investigating the mechanism of protective immune responses elicited by the BCG and a continuous effort in refining TB mouse model as the first line of screening tool for the selection of new TB vaccine candidates.

Bretscher^18,19^, proposed a concept that the dose of BCG given to children is too high leading to a mixture of TH1 and TH2 responses, which reduces the level of resistance by the vaccine. In contrast, low dose of BCG will induce a TH1 responses. Following on the Bretscher’s concept, Gruppo V and Orme I in 2002^20^ vaccinated BALB/c mice with BCG at 10^7^, 10^5^ and 10^3^ CFU and challenged them with H37Rv. While it was found that high doses of BCG could induce TH2 responses, but it did not interfere with the resistance to H37Rv. The study^20^ mainly used H37Rv and BALB/c mice, whereas in our study we have used two different mouse strains and two different *Mtb* strains with various BCG doses of 10^5^, 10^4^, 3000, 300 and 30 CFU. Our study was designed to accommodate and address varied observations such as 1. To evaluate protection of various doses of BCG in BALB/c and CB6F1 mice when infected with H37Rv or HN878 and to understand the protective cellular immunity induced by various BCG doses; 2. For dose sparing of BCG vaccine and the refinement of TB mouse model, we were interested to evaluate protective responses of BCG dose at 3000 CFU. BCG dosage at 1–4 × 10^5^ CFU is generally given to infants, preferably at the time of birth. The commonly used BCG dose in mice is also at 1–4 × 10^5^ CFU/mouse. If the body weight of an infant and an adult mouse is considered, the BCG dose at 3** × **10^5^ CFU given to a mouse would be around 150 times in excess compared to the BCG dose in an infant. Therefore, most of the research conducted in mice using BCG dose at 1–4 × 10^5^ CFU would be around two Log higher compared to a single human dose and the immune parameters measured would fall under the high dose BCG responses. According to the study by Bretscher^21,22^ high dose of BCG would skew the immune response more towards the mixture of TH1 and TH2. Therefore, it was pertinent to evaluate the protection and immune responses of 3000 CFU in mice, an equivalent dose of BCG at 1–4 × 10^5^ CFU in infants; 3. For the BCG dose sparing in preclinical studies, concerns were raised in 2016^23^ for the shortage of global BCG vaccine and the published study in 2019^24^ highlighted the effect of shortage with the rise of TB meningitis in children. The shortage in global BCG supply has also led to review of the established treatment guidelines in rationing of the administration of BCG for bladder cancer^25^.

Considering the principle of 3Rs, our in vivo experiments were designed with optimal number of mice per group in order to achieve sufficient statistical power which was demonstrated from the results of positive control group of each experiment. Although the data generated from the HN878 challenge experiment in BALB/c and CB6F1 mice and immunogenicity evaluation were from single experiment, respectively and we are confident that these data are the true representation of the study. The variable parameters between the experiments are kept at minimum; use of same batch of BCG WHO reference reagents, same batch of frozen aliquots for *Mtb* challenge; similar timepoints for testing or harvesting mice for TB determination (set times in the morning) and consistent routine husbandry schedule. Such checks of variable parameters have proved to show similar results in most of our mice experiments (also our published^26^ and unpublished data from our group) as well as from the duplicate experiments of H37Rv challenge experiment in BALB/c and CB6F1 mice.

Results from the Log_10_ CFU/lungs data demonstrate that the lowest dose at 30 CFU failed to protect both strains of adult mice in the lungs when infected via aerosol route with either H37Rv or HN878. However, in the study conducted by Kiros et al., 2010^22^*,* BALB/c mice were vaccinated with BCG Danish at 30 CFU protected mice. However, in that study^22^, new-born mice and H37Rv infection via intravenous route were used, whereas in our study, adult mice and two strains of *Mtb* (H37Rv and HN878) infection via aerosol route were used. In our study, the protection against TB was influenced by the type and route of infection of *Mtb* strain for the BCG doses at 3 × 10^5^ to 300 CFU in both strains of mice. H37Rv infected BALB/c and CB6F1 mice displayed significant protection in the lungs when vaccinated with BCG doses at 3** × **10^5^ to 300 CFU. However, for HN878 infected mice, the lowest protective BCG dose in the lungs of BALB/c mice was BCG at 3000 CFU and for CB6F1 mice it was 300 CFU. Furthermore, as per the increasing doses of BCG, a decremental trend in the TB burden (Log_10_ CFU/lungs) was observed in the lungs of BALB/c and CB6F1 mice when infected with HN878 whereas, this trend was not seen for H37Rv infected BALB/c and CB6F1 mice. Together these data have shown the importance of choosing the combination of mouse strains and the choice of TB strains, such as BALB/c mice infected with H37Rv or HN878 showed ρ = 0.0001 or ρ = 0.005 significant difference in the lungs of BCG dose at 3 × 10^5^ CFU, respectively. Whereas, in the CB6F1 mice infected with either H37Rv or HN878 displayed similar protection in the lungs of BCG dose at 3 × 10^5^ CFU. In addition, BCG dose 3000 CFU showed ρ = 0.0001 or ρ = 0.05 significant difference in the lungs of BALB/c infected with either H37Rv or HN878, respectively and no protection of BCG 300 CFU in the BALB/c mice when infected with HN878. Therefore, use of both strains of mice BALB/c and/or CB6F1 infected with HN878 *Mtb* strain is an interesting combination to study and should be considered for the pre-clinical TB vaccine testing in mice.

CD4 T cells play a central role for the protective immunity against TB, as described by several human studies and animal models of TB^27–29^. The important findings from Sallusto and her colleagues^30–32^ indicated that there are two important subsets of memory T cells—TEM and central memory T cells (TCM). Differentiation of these memory T cells is based on the clear phenotypic characteristics—TEM are CD44^hi^ CD62L^lo^ CCR7^lo^ while, TCM are CD44^hi^ CD62L^hi^ CCR7^hi^. However, recent evidence depicts a complex interplay and diverse functionality under the broad subsets of memory T cells^33^. From a published study^34^, TEM CD4^+^ CD44^hi^ CD62L^lo^ expressing IFNγ/IL2/TNFα cells in the spleen of BCG vaccinated mice expanded post *Mtb* infection and these BCG-specific TEM cells shown to have a strong association with the protection against *Mtb* infection^34^. Therefore, the data in this study for the analysis of TEM cells were focussed post BCG vaccinated and not post *Mtb* infection. When we examined TEM CD4^+^ CD44^hi^ CD62L^lo^ expressing IFNγ/IL2/TNFα in the spleens of BALB/c and CB6F1 mice, the TEM cells were absent in the BCG 300 and 30 CFU groups for BALB/c mice, while in CB6F1 mice TEM cells were absent for BCG 30 CFU group only. The correlation of TEM cells expressing IFNγ/IL2/TNFα for BCG dose groups and the correspondent protection data (Log_10_ CFU/lungs) in the lungs of BALB/c and CB6F1 mice, showed significant correlation for both strains of mice when infected with HN878, but no correlation when infected with H37Rv. This is an interesting finding to suggest that may be the TEM cells expressing IFNγ/IL2/TNFα are valuable in BALB/c or CB6F1 mice for protection against HN878 infection.

Interestingly, the protection data (Log_10_ CFU/lungs) in the lungs of BALB/c mice for BCG vaccinated groups significantly correlated with TEM cells expressing IFNγ/TNFα. Although, for the BCG 300 CFU group in BALB/c mice, TEM cells were not detected, and yet when infected with H37Rv, the protection was equivalent to BCG 3 × 10^4^—3000 CFU groups, whereas BCG at 300 CFU group could not protect against HN878 infection. This is an interesting observation, if the protection in H37Rv infected BALB/c mice was not due to double or triple cytokine expressing CD4 TEM, this may suggest that either the protection was from the TEM independent mechanism or due to strain heterogeneity of *Mtb* or the presence of virtually undetected TEM. BCG at 300 CFU group in BALB/c mice is the lowest protective dose which is influenced by the type of *Mtb* infecting strain and would be an interesting dose of choice for research to understand BCG efficacy against different *Mtb* strains.

Examination of the cytokine responses in the M7 stimulated cells isolated from the lungs and spleen revealed a distinct pattern of cytokine/chemokine between BALB/c and CB6F1 mice. The cytokines and chemokines concentrations have undergone robust data analysis: first, the concentration of cytokines/chemokines were interpolated from the linear part of the 5-parameter standard curve and second, the interpolated concentrations from the supernatants of M7 stimulated cells were subtracted with those of unstimulated cells supernatant and these concentrations were used for the data analysis. IFNγ is critical for the TB protection and BCG is the only vaccine whose protective immunity is believed to be depended on the induction of CD4^+^ T cells that produce IFNγ, which in turn activates macrophages to kill *Mtb*^35,36^. As we expected, IFNγ responses in the supernatants of M7 stimulated splenocytes and lung cells of BCG groups showed correlation with the ex vivo IFNγ responses for both strains of mice. The IL2, GM-CSF and CXCL13 responses from the supernatants of stimulated splenocytes were significantly inversely correlated with the *Mtb* burden in the lungs of CB6F1 mice infected with either H37Rv or HN878. This may suggest that the low concentration of detected IL2, GM-CSF and CXCL13 in the spleen can be used as an immunological marker for CB6F1 mice in preclinical TB vaccine testing. However, in BALB/c mice, IL2, GM-CSF and CXCL13 responses either from the supernatants of stimulated splenocytes or lung cells were not correlated with the *Mtb* burden in the lungs of infected mice. Surprisingly, the concentrations of cytokine/chemokine in the lowest dose group, 30 CFU which failed to protect *Mtb* infected BALB/c and CB6F1 mice followed the cytokine/chemokine responses almost like BCG 300 CFU and not the control group. High dose of BCG has been implicated to skew immune responses in the mixture of TH1/TH2, but we could not detect any responses for IL4 in BALB/c or CB6F1 mice.

This study has provided some interesting suppositions and questions: 1. Though there were no protection in the lungs of BALB/c or CB6F1 mice after BCG vaccination with 30 CFU, we were surprised to find that the cytokine/chemokine responses were not like the control group. In fact, they were more like those in the BCG 300 CFU group, which indeed protected CB6F1 and BALB/c mice infected with H37Rv; 2. Animal studies commonly use BCG dose at 3 × 10^5^ CFU and have shown to persist in mice for 18 months^34^, the protection against *Mtb* in these mice is suggested to be from constant priming of TEM by the presence of live BCG^37^. Does this depicts that the loss of protection in the lowest dose at 30 CFU is due to the waning of BCG which subsequently ceases constant priming of TEM cells or is it possible that BCG dose at 30 CFU is so low that the frequency of TEM cells failed to establish at an optimum level to fight against *Mtb* infection? 3. Depending on the research question, 300 CFU would be an interesting BCG dose to study in BALB/c and CB6F1 mice: BCG dose at 3 × 10^5^ CFU persists in organs for many months in mice and use of BCG dose at 300 CFU may shorten the experimental time and effectively provide a realistic time scale to speed up a waning experiment.

Finally, we conclude that BCG dose at 3000 CFU per mouse, a human equivalent dose, protects BALB/c and CB6F1 mice when infected with H37Rv or HN878 which also is a realistic window to test differences in the protection of the new TB vaccine against the benchmark BCG vaccine. The combination of *Mtb* and mouse strain is an important parameter for evaluating new TB vaccine against different clinical isolates and must be considered. The spleen IL2, GM-CSF and CXCL13 alongside lungs IFNγ from the supernatants of stimulated splenocytes or lung cells may be used as an important immunological marker for a preclinical TB vaccine testing in CB6F1 mice.

## Methods

### Ethics and animals

All animal procedures were performed in accordance with the UK Home Office (Scientific Procedures) Act 1986; under appropriate licences, the study protocol was approved by the NIBSC Animal Welfare and Ethics Review Body.

BALB/c and CB6F1 mice, female, 8 weeks old and with approximately 15–20 gm body weight were obtained from the SPF facilities at the Charles River UK Ltd. All animals were housed in an appropriate BSL2 (BCG vaccinated) and BSL3 (TB infected) containment facilities at NIBSC. Mice were checked daily throughout the experiment by trained animal technicians and this frequency could increase if any adverse reactions were observed. All mice were weighed before TB infection and subsequently weighed weekly post infection until scheduled experiment termination by schedule 1, method of cervical dislocation followed by confirmation of death. No adverse effect or severe weight loss (more than 20% body weight) was observed in any mice for the entire duration of the study. The study was performed in compliance with the ARRIVE guidelines.

### Study groups

The study was divided into two parts: 1, H37Rv or HN878 challenge for BALB/c and CB6F1 mice; five mice per group were vaccinated via intradermal route with different doses of BCG Danish 1331 (3 × 10^5^, 3 × 10^4^, 3000, 300 or 30 CFU/mouse) and sterile saline was administered to the control group. Four weeks post vaccination, all groups (five mice/group) were infected with *Mtb* H37Rv or HN878 via aerosol route. BALB/c and CB6F1 mice infected with H37Rv had BCG at 3 × 10^5^, 3 × 10^4^, 3 × 10^3^, 300 and 30 CFU, whereas in HN878 infected BALB/c and CB6F1 mice, all BCG vaccinated groups except 3 × 10^4^ CFU were selected. 2, Immunogenicity evaluation; BALB/c and CB6F1 mice received different doses of BCG except 3 × 10^4^ CFU (3 × 10^5^, 3000, 300 or 30 CFU/mouse) via the intradermal route. At 6 weeks post vaccination, the lungs and spleen were harvested from all mice for the preparation of single cell suspension in a subsequent immunological analysis.

### BCG vaccination and Mtb challenge

BCG vaccination for BALB/c and CB6F1 mice for H37Rv challenge experiment was performed using an expired commercial preparation of Staten Serum Institute BCG Danish 1331 vaccine stored at -20˚C. Even though the BCG Danish 1331 was expired, lyophilised BCG was equally viable to use in mice as we demonstrated by plating out serial dilutions of BCG in sterile saline to obtain approximately 6 × 10^6^ CFU/ml (Supplementary Table 1). However, for HN878 challenge experiment and immunogenicity evaluation, the WHO Reference Reagent for BCG vaccine of Danish 1331 sub-strain preparation (NIBSC code 07/270^16^; https://www.nibsc.org/products/brm_product_catalogue/detail_page.aspx?catid=07/270) stored at − 20 °C was used (Supplementary Table 1). The two vaccines i.e. Staten Serum Institute BCG Danish 1331 vaccine and WHO Reference Reagent for BCG vaccine of Danish 1331 are the same strains of BCG. As per the WHO’s request to establish BCG reference reagent, Statens Serum Institute in Denmark donated the lyophilised, sterile pre-filled ampoules of BCG Danish 1331 strain to NIBSC^16^ (NIBSC code: 07/270). Subsequent study demonstrated that the WHO reference reagent for BCG Danish 1331 (NIBSC code: 07/270) and Staten Serum Institute BCG Danish 1331 exhibit similar protection in mice when infected with *Mtb*^26^. Therefore, the BCG Danish 1331 strains used in this study are from the same source^16^ and demonstrated to be similar in protection against *Mtb* infection^26^. Our cultural viable count assay showed when lyophilised BCG preparations stored at − 20 °C their viability retained for long term storage. Lyophilised BCG preparations were reconstituted and diluted in sterile saline to obtain approximately 6 × 10^6^ CFU/ml. Subsequent dilutions (Supplementary Table 1) were prepared in sterile saline to obtain various doses of 3 × 10^5^ to 30 CFU/mouse, administered in 2 × 25 μL injections by bilateral intradermal route, whereas sterile saline was administered in the control group. All BCG doses (3 × 10^5^ to 30 CFU) were checked by plating in duplicate onto 7H11 agar plates (DIFCO) supplemented with Oleic Acid Dextrose Catalase (OADC, BD) and CFU/ml were estimated at 4 weeks post incubation at 37 °C (Supplementary Table 1).

For low-dose aerosol infections, H37Rv or HN878 frozen stocks (PBS containing 50% glycerol) were diluted in sterile distilled water to approximately 5–7 × 10^6^ CFU/ml (Supplementary Table 2) and placed in a Glass nebulizer‐venturi (Glas‐Col)^38,39^. The aerosol generated was transferred to an infection chamber for whole body exposure (Walkers, UK), which operates on a similar concept as Madison infection chamber^38^ .The mice were exposed to an aerosol infection in which live bacilli were deposited in the lungs of each mouse (expected ~ 100 CFU/ lungs). Confirmation of low dose infection for H37Rv and HN878 challenge experiment was performed by necropsied five BALB/c mice (Supplementary Table 2) on the same day of challenge. Lung lobes were removed, homogenised and serially diluted samples were plated in duplicate onto 7H11 agar (DIFCO) supplemented with OADC (BD). Bacterial colonies were enumerated 3–4 weeks post incubation at 37 °C. Upon termination of the experiment at 4 weeks post challenge, mice were necropsied, the spleen and lungs were removed, homogenised and serially diluted samples were plated in duplicate onto OADC-supplemented 7H11 agar plates. The plates were incubated at 37 °C and bacterial colonies were enumerated after 3–4 weeks.

In our study, we have not used a specialised 7H11 growth medium to facilitate *Mtb* over BCG growth, as a published study^40^ have shown the absence of residual BCG in the samples of BCG subcutaneous group post 6 weeks of BCG vaccination. In addition, another published study^37^ have shown the growth of intradermal BCG in the lungs of mice was sporadic starting from 4 weeks post BCG vaccination up to 22 weeks after which BCG was undetected. The data in Fig. 1 is 8 weeks post intradermal BCG vaccination (4 weeks of BCG vaccination followed by 4 weeks of *Mtb* infection) and therefore, the growth of BCG (if any) is unlikely to affect *Mtb* burden in the lungs of infected mice.

### The lung cells and splenocytes preparation

For the immunogenicity evaluation, the lungs and spleen were harvested for isolating single cell suspension. For the lung cells preparation, harvested lung lobes were minced and agitated for 1 h at 37 °C in supplemented DMEM medium containing 50 U/ml collagenase I (Gibco) and 10 U/ml DNase II (Sigma), followed by passing through a 40 µm cell strainer. Isolated lung cells were washed and resuspended in supplemented DMEM at 1 × 10^7^ cells/ml for use in the subsequent assays.

For splenocytes isolation, the spleen was mashed through a 40 µm cell strainer, washed at 300 g for five minutes and resuspended at 1 × 10^7^ cells/ml in the supplemented Dulbecco’s Modified Eagle’s Medium (DMEM, Sigma) containing 10% heat inactivated foetal calf serum (Biosera, UK) and 2% penicillin/streptomycin (Gibco) for use in the subsequent assays.

### Mycobacterial antigens (M7 protein cocktail)

The M7 protein cocktail, which is made of a pool of seven immunogenic recombinant mycobacterial proteins—Rv1886c (Ag85B), Rv0251c (hsp20), Rv0287 (TB9.8), Rv0288 (TB10.4), Rv3019c (TB10.3), Rv3763, Rv3804c (Ag85A) (Lionex, GmbH, Germany) and stored at − 80 °C, was used in assays where cells required in vitro stimulation. The recombinant proteins included in the M7 protein cocktail are present in the BCG and/or *Mtb*. Each protein concentration in the cocktail is 50 µg/ml and for stimulating cells M7 cocktail was diluted to achieve a final concentration of 2 µg/ml for each protein. M7 protein cocktail can be purchased from the NIBSC website.

### ELISPOT

Approximately 2 × 10^5^ splenocytes or lung cells were incubated in duplicate in 96-well filter plates (MSIPS4510 Millipore, Ireland) with or without M7 protein cocktail (final conc. 2 µg/ml), or concanavalin A (10 µg/ml, Sigma) as a positive control for approximately 16 h at 37 °C. The frequency of IFNγ secretors was detected by the AID ISPOT (ELR08IFL) reader (AID Autoimmun Diagnostika, Germany), as per manufacturer's instructions.

### Flow cytometry for surface markers and intracellular cytokines

The flow cytometry analyses on splenocytes were performed as previously described^34^. In brief, splenocytes were cultured at 1 × 10^7^ cells/ml with 2 µg/ml of M7 protein cocktail, 1 µg/ml of anti-CD28 (clone: 37.51, BD Biosciences) for 2 h at 37 °C under 5% CO_2_, after which 10 µg/ml Brefeldin A (Sigma) was added for a further incubation of 14 h. Subsequently, cells were washed at 300 g for five minutes and surface stained with a combination of pre-titrated monoclonal antibody conjugates: CD90.2-eFluor 450 (clone: 53-2.1); CD27-PerCP-eFluor710 (clone: LG.7F9, both Life Technologies, UK); CD62L-FITC (clone: MEL-14); CD4-APC-H7 (clone: GK1.5, both BD Biosciences); CD8-Alexa Fluor 700 (clone: 53-6.7); CD44-Brilliant Violet 785 (clone: IM7); and Zombie Aqua Fixable Viability Dye (all BioLegend). Cells were then washed, fixed, permeabilised and stained intracellularly using BD Cytofix and BD Cytoperm (BD Bioscience) as per manufacturer’s instructions, with a combination of: IL17a-PE (clone: TC11-18H10BD, BD Biosciences); IFNγ-PE-Cy7 (clone: XMG1.2); IL2-APC (clone: JES6-5H4); and TNFα-BV605 (clone: MP6-XT22) all from BioLegend. Cells were analysed immediately after final staining. Data were acquired using a SORP LSR Fortessa (BD Bioscience), utilizing a 532 nm laser for PE and PE-Cy7, and analysed on Flowjo v.10.1 software (BD Biosciences). All analyses were gated (Gating strategy in Supplementary Fig. 2) on a minimum of 100,000 live lymphocytes. Compensation was performed using UltraComp eBeads (Life Technologies) according to the manufacturer’s instructions. Fluorescence minus one control were used to set gates for cytokine analyses.

### Multiplex cytokine/chemokine bead assays

The lung cells and splenocytes at ~ 2 × 10^6^ cells/ml were stimulated with or without M7 cocktail (final conc. 2 µg/ml) and incubated at 37 °C under 5% CO_2_ for 3 days. Supernatants were harvested and cytokine/chemokine bead assays was performed using BioRad’s Bio-Plex pro mouse cytokine 34 or 31-plex assay. BioRad’s 34-plex was used for the BALB/c mice and, due to the availability of the reagent kit, 31-Plex was used for CB6F1 mice (Table 3). All multiplex magnetic bead assays were performed according to the BioRad’s protocol and data were acquired using Luminex 200 LX equipment. Concentrations of cytokine/chemokine were interpolated using manufacturer’s provided standard cocktail in the kits. For the data analyses, an estimated concentration (pg/ml) of cytokine/chemokine were interpolated from the linear part of the 5-parameter standard curve. Furthermore, the interpolated concentrations from the supernatants of stimulated cells were subtracted with those of unstimulated cells supernatant and these concentrations were used for the data analysis (Fig. 3 and Supplementary Table 3).

### Statistical analyses

All data were analysed with the GraphPad Prism 8 software (Graph Pad, USA) using one- or two- way ANOVA with Dunnett’s or Tukey’s post-test (three-treatment groups), respectively. Mycobacterial CFUs were Log_10_ transferred before comparison. Correlation coefficients were assessed using Pearson’s two-tailed correlation test with 95% confidence interval. Differences with a ρ value < 0.05 were considered and denoted with, *ρ < 0.05, **ρ < 0.005, ***ρ < 0.0005, ****ρ < 0.0001.

## Supplementary Information

Supplementary Information.
