# Supplementary material for: Efficacy and immunogenicity of different BCG doses in BALB/c and CB6F1 mice when challenged with H37Rv or Beijing HN878
Source: Sci Rep. 2021 Dec 2;11:23308. doi: 10.1038/s41598-021-02442-5 (PMC8639814; doi:10.1038/s41598-021-02442-5)
Supplement: Supplementary file 1 — Supplementary Information. [file 41598_2021_2442_MOESM1_ESM.pdf]

Efficacy and immunogenicity of different BCG doses in BALB/c and CB6F1 mice when challenged with H37Rv or Beijing HN878

Bhagwati Khatri [1]*, James Keeble [1], Belinda Dagg [1], Daryan A. Kaveh [2], Philip J. Hogarth [2] and Mei Mei Ho [1]

[1]Bacteriology Division, National Institute for Biological Standards and Control, South Mimms, Potters Bar, Hertfordshire, EN6 3QG, United Kingdom.

[2]Department of Bacteriology, Animal and Plant Health Agency, Addlestone, Surrey, KT15 3NB, United Kingdom.

***bhagwati.khatri@nibsc.org**

Supplementary Information

Supplementary Table 1: Enumeration of BCG CFU/ml

**Expected BCG CFU/ml**	**SSI BCG Danish 1331 strain CFU/ml for H37Rv challenge experiment**		**WHO RR BCG Danish 1331 strain (NIBSC code 07/270) CFU/ml for HN878 challenge experiment**	
	**Average CFU/ml (+ SD) enumeration using duplicate 7H11 plates (technical replicates)**	**Actual dose of BCG CFU per mouse (50µl given by ID route)**	**Average CFU/ml (+ SD) enumeration using duplicate 7H11 plates (technical replicates)**	**Actual dose of BCG CFU per mouse (50µl given by ID route)**
**$6x10^6$ CFU/ml**	4.3 (+0.57) x 10^6 CFU/ml	2.15 x 10^5 CFU	2.4 (+0) x 10^6 CFU/ml	1.2 x 10^5 CFU
**$6x10^5$ CFU/ml**	3.75 (+0.70) x 10^5 CFU/ml	1.88 x 10^4 CFU	Excluded from the exp.	Excluded from the exp.
**$6x10^4$ CFU/ml**	3.85 (+0.35) x 10^4 CFU/ml	1930 CFU	2 (+0.28) x 10^4 CFU/ml	1000 CFU
**6000 CFU/ml**	2200 (+282) CFU/ml	110 CFU	3000 (+700) CFU/ml	150 CFU
**600 CFU/ml**	240 (+40) CFU/ml	12 CFU	340 (+14) CFU/ml	17 CFU

Supplementary Table 2: Enumeration of CFU/ml for Infection inoculum (technical replication from the duplicate 7H11 plates) and the lungs of 5 mice (Biological replicates) on the same day of aerosol infection with H37Rv and HN878.

	**H37Rv challenge**	**HN878 challenge**
**Expected challenge inoculum**	5.45 x 10^6 CFU/ml	7 x 10^6 CFU/ml
**Average CFU/ml (+ SD) enumeration using duplicate 7H11 plates**	3.6 (+0.85) x 10^6 CFU/ml	4 (+0.91) x 10^6 CFU/ml
**Average CFU in the lungs of five BALB/c mice (Av. CFU + SD)**	71 (+64) CFU	144 (+63) CFU

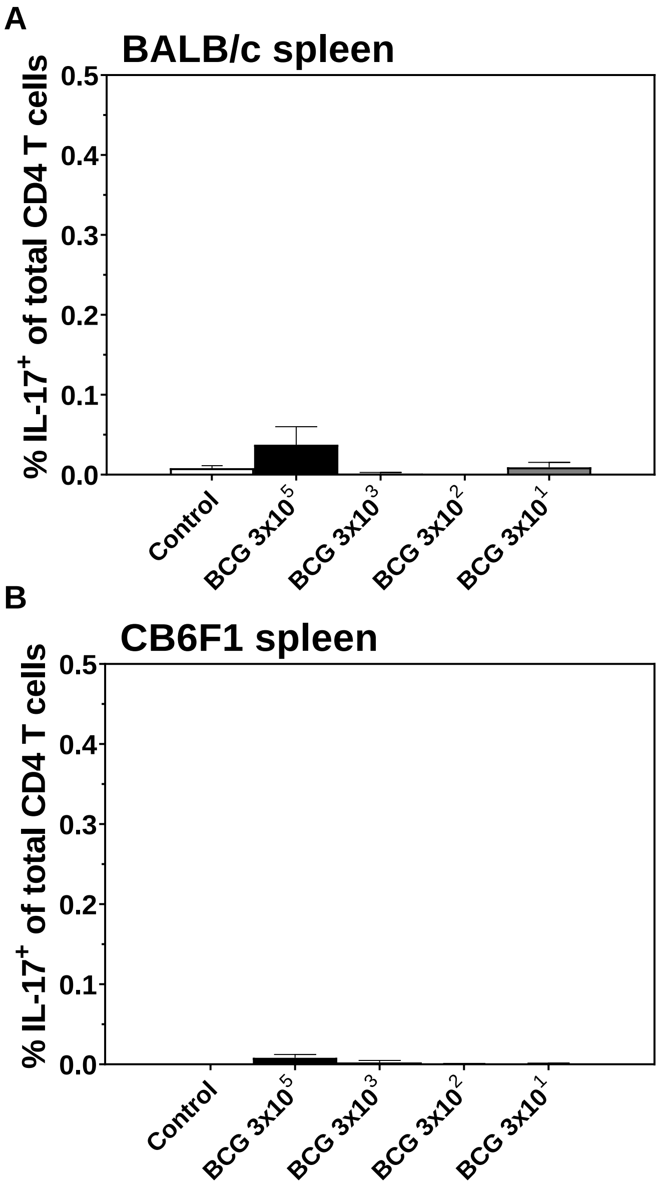

Supplementary Figure 1: Vaccination induced resident multifunctional CD4$^+$ cells expressing IL17a in the spleen of BALB/c and CB6F1 mice. Using flow cytometry, the frequency of multifunctional CD4$^+$CD44^{hi}CD62L^{lo} cells expressing IL17a at six weeks following BCG immunization. A, splenocytes from BALB/c (5 mice per group) and B, CB6F1 (5 mice per group) mice. Control and BCG groups - 3x10^5, 3000, 300 and 30 CFU were isolated, stimulated with M7 protein cocktail and stained by ICS. Data is the representative of one independent experiment for both strains of mice. Bars represent mean % frequency of cells of indicated T cell phenotype as a % of total CD4+ cells. Two-way ANOVA, Tukey's multiple comparison was performed and found no significant difference between the BCG vaccinated groups when compared to the control group in the spleen of BALB/c and CB6F1 mice.

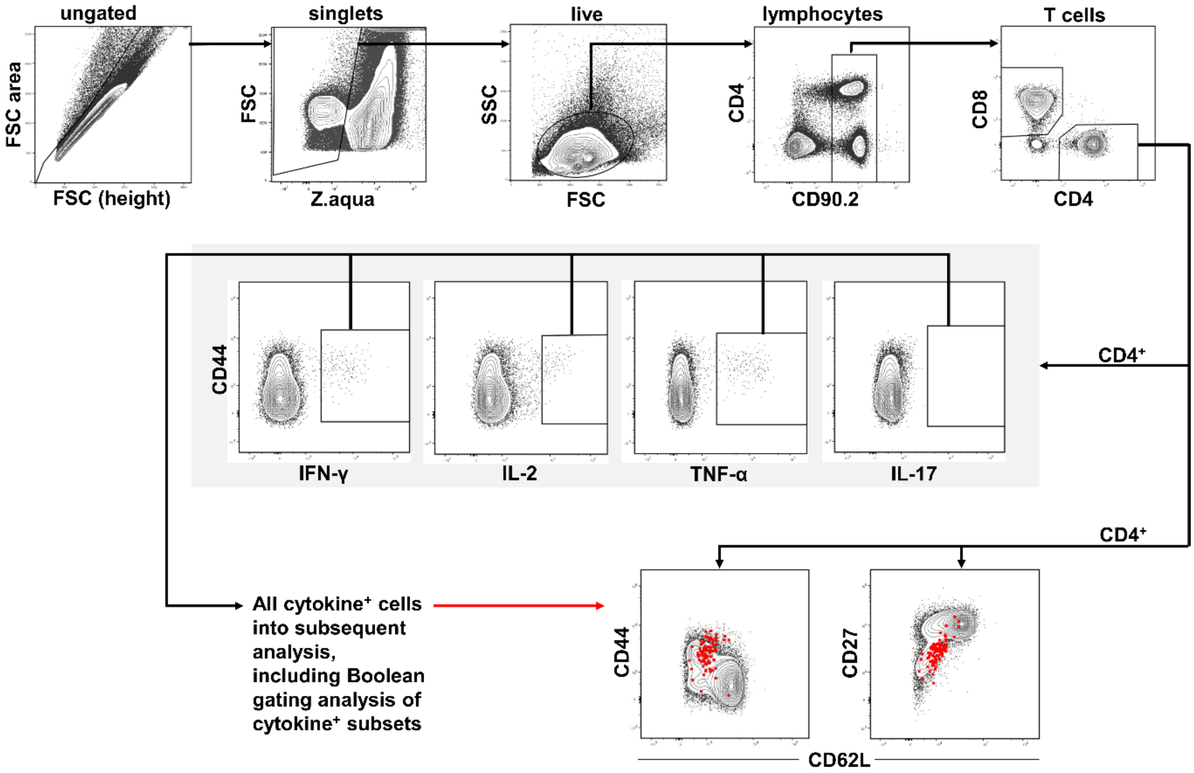

Supplementary Figure 2: Flow cytometry gating strategy. Gating strategy used for identification of CD4 T cells producing IFNγ, TNFα, IL2, and IL17 alone or in any combination. Cells were gated on singlets followed by live cells and then lymphocytes. T cells were identified as CD90.2$^+$, before gating on CD4$^+$ cells. Antigen-specific CD4 T cells were then identified by their production of IFNγ, TNFα, IL2 or IL17. Boolean gating was then used to identify all cells producing any combination of one or more of these cytokines (termed cytokine+) as well as the individual cytokine+ CD4 T cell subsets defined by the different simultaneous combinations. Cytokine+ cells (large red dots) were also evaluated for their surface expression of CD62L in combination with either CD44 and CD27 as compared to the total CD4 T cell population (overlaid contour plot).

Supplementary Table 3: Cytokine/chemokine values in pg/ml using Bio-plex Manager 6.1 software. Note: All concentrations in pg/ml were obtained from the linear part of the 5P standard curve using Bio-Plex Manager 6.1 software. The concentrations (pg/ml) in the table represent the result after subtracting concentrations of cytokines/chemokines in the supernatant of M7 stimulated cells with those from unstimulated cells. Blue highlighted cells in the table shows significance ($p \leq 0.05$) when compared to the control group.

Cytokine/Chemokine	BALB/c - Spleen					CB6F1 - Spleen					BALB/c - Lungs					CB6F1 - Lungs				
	Control	3.E+05	3000	300	30	Control	3.E+05	3000	300	30	Control	3.E+05	3000	300	30	Control	3.E+05	3000	300	30
CXCL13	168.87	170.69	150.70	24.17	105.32	69.53	41.98	35.11	53.40	84.86	121.87	114.40	132.95	48.17	151.29	25.81	-1.81	47.28	33.47	8.96
CCL27	153.97	182.53	115.66	23.64	-13.98	119.39	134.62	57.62	193.54	160.54	72.36	153.76	144.39	-6.37	113.67	331.29	243.39	261.43	428.30	69.39
CXCL5	113.85	119.82	100.28	3.52	28.12	38.53	39.27	28.04	57.05	60.51	207.05	937.72	194.78	121.61	70.65	579.98	463.82	439.14	456.82	303.55
CCL11	2.83	2.63	1.93	0.00	0.00	1.27	1.05	0.43	1.33	1.34	2.66	4.41	2.97	0.67	1.51	2.28	2.63	1.89	2.52	1.21
CCL24	0.00	0.00	0.00	0.00	0.00	0.00	0.00	0.00	0.00	0.00	-267.46	-248.19	-432.30	-157.59	8.63	-741.23	-449.21	-370.47	-313.01	-344.96
CX3CL1	5.05	5.05	2.90	-24.27	-41.30	7.72	18.22	-0.53	8.76	13.38	4.07	13.02	9.78	-3.65	0.00	6.93	6.26	7.98	8.26	1.49
GM-CSF	8.26	8.27	5.70	-26.10	-44.58	7.02	14.44	12.19	13.57	8.14	18.54	47.75	15.48	8.33	7.89	60.10	50.55	43.36	37.39	29.06
CCL1	0.95	27.32	13.36	-26.82	-46.05	1.00	9.91	5.23	11.82	2.30	1.41	129.23	21.17	0.10	0.42	2.15	32.21	14.79	30.26	1.40
IFNγ	6.58	728.58	689.73	5.89	6.32	99.98	590.08	362.33	557.85	121.91	2.75	85.89	9.99	-0.30	2.40	2.59	15.92	6.68	8.94	1.27
IL1β	2.83	5.75	5.75	-28.63	-19.34	11.02	24.72	13.81	13.81	9.00	11.56	30.39	12.83	0.00	5.13	6.87	12.87	8.91	18.43	10.73
IL2	1.62	43.10	25.44	-26.46	-45.74	0.56	16.99	10.81	8.80	1.01	0.92	34.06	6.22	0.43	0.23	0.94	13.28	3.76	3.68	0.29
IL4	0.31	0.94	0.84	-27.09	-45.89	3.50	0.08	-3.90	2.61	3.04	0.86	1.38	0.93	0.33	0.69	1.91	1.24	1.90	4.44	-0.99
IL6	387.64	919.72	596.51	84.30	169.00	1029.57	1631.93	1410.87	1815.13	1466.01	1140.35	8510.27	1400.92	492.99	466.87	1783.44	1392.55	918.52	1180.71	601.25
IL10	59.19	92.71	78.71	-19.40	-6.10	150.47	187.87	138.24	196.91	202.62	39.98	72.35	50.69	1.79	28.33	0.00	0.00	0.00	0.00	0.00
IL16	-197.70	-177.85	-102.25	-66.36	209.92	37.42	24.81	-105.50	-99.39	-48.73	-31.22	50.44	-32.26	-37.25	-4.06	56.42	72.80	107.21	97.58	100.09
IP10	39.13	54.81	56.82	-9.62	28.46	274.26	470.84	301.07	694.21	396.45	37.11	174.33	29.61	-6.00	61.84	235.90	321.09	222.40	265.10	146.99
CXCL11	31.17	28.13	22.89	-20.59	-24.73	84.58	112.96	80.61	139.18	130.62	29.37	349.88	44.17	5.09	26.47	256.82	196.65	207.51	273.50	72.12
CXCL1	1436.42	1098.90	705.91	202.45	313.63	788.70	790.92	609.16	812.23	994.41	5523.74	6022.01	7135.67	2271.40	3078.96	13393.78	7275.09	7233.32	9781.89	4663.81
CCL2	200.71	87.40	66.79	-21.94	-34.80	40.57	66.87	56.49	63.71	64.64	973.77	9086.06	980.94	642.08	359.00	1935.26	1739.76	1620.55	1584.96	931.70
CCL8	0.00	0.00	0.00	0.00	0.00	not included in the multiplex					0.00	0.00	0.00	0.00	0.00	not included in the multiplex				
CCL7	45.90	14.05	12.03	-25.06	-41.80	3.88	5.09	4.62	6.44	7.31	110.95	1190.52	244.05	34.89	65.62	184.52	141.83	127.11	197.90	71.81
CCL12	0.00	0.00	0.00	0.00	0.00	0.00	0.00	0.00	0.00	0.00	0.00	0.00	0.00	0.00	0.00	0.00	0.00	0.00	0.00	0.00
CCL22	537.69	611.09	469.72	110.87	277.64	135.22	235.51	177.95	345.22	321.69	588.50	5473.11	732.04	263.57	279.16	392.31	206.03	153.68	196.50	137.65
CCL3	1837.50	1977.79	1098.59	372.64	575.69	2264.52	1692.59	1747.28	2735.61	4499.99	7015.16	7784.34	7322.46	2586.61	5050.60	1334.41	1719.81	1869.16	4748.09	1195.80
CCL4	893.56	977.03	638.52	112.99	260.74	628.36	808.47	696.04	877.43	799.87	1144.69	2511.22	1738.81	547.90	632.20	1739.16	1161.62	784.57	933.29	546.65
CXCL2	4265.26	3790.41	2301.22	722.75	1154.43	not included in the multiplex					56136.94	293922.42	627063.28	8264.20	17789.77	not included in the multiplex				
CCL20	0.00	0.00	0.00	0.00	0.00	0.00	0.00	0.00	0.00	0.00	-2.73	0.75	-0.02	-1.84	1.22	0.00	0.00	0.00	0.00	0.00
CCL19	8.75	13.93	13.11	-23.60	-38.04	17.10	26.06	15.23	28.89	36.90	6.48	11.26	2.92	-0.66	10.33	27.32	22.77	-3.26	35.77	14.80
CCL5	146.31	420.80	283.45	41.33	97.91	495.87	653.70	514.05	630.39	531.09	262.78	1237.95	347.81	157.65	202.02	801.52	654.37	515.44	643.18	315.44
CXCL16	6.17	6.53	5.30	0.00	OOR<	6.65	6.08	4.00	5.02	7.22	24.39	161.01	40.44	5.53	11.90	48.66	32.77	30.15	30.49	14.51
CXCL12	23.71	27.87	26.51	-20.35	-20.92	87.47	86.05	82.06	99.65	118.72	33.88	49.16	25.11	10.30	29.75	76.90	81.14	61.46	77.82	53.91
CCL17	8.97	9.22	7.24	-25.09	-42.90	13.49	12.64	8.51	16.35	15.39	17.89	73.16	12.23	1.17	8.03	54.45	31.00	28.31	40.99	21.76
TNFα	32.93	23.15	9.93	-25.60	-42.25	11.59	16.48	7.60	12.01	10.51	94.35	510.31	139.07	32.61	23.54	26.56	19.87	13.48	12.91	7.90
CCL25	0.00	0.00	0.00	0.00	0.00	not included in the multiplex					0.00	0.00	0.00	0.00	0.00	not included in the multiplex				

Supplementary Table 4: Correlations between IFNγ and other cytokines in the supernatants of M7 stimulated splenocytes and lung cells from mice vaccinated with BCG vaccine.

	BALB/c lungs IFNγ			CB6F1 lungs IFNγ		
Lungs cytokines	Pearson r	P (two tailed)	P value summary	Pearson r	P (two tailed)	P value summary
BCA-1 CXCL13	0.06569	0.9164	ns	-0.3095	0.6123	ns
CCL27	0.57	0.3158	ns	0.3098	0.612	ns
CXCL5	0.991	0.001	**	0.1774	0.7753	ns
CCL11	0.8178	0.0908	ns	0.7418	0.1513	ns
CCL24	-0.1762	0.7768	ns	0.2292	0.7107	ns
CX3CL1	0.7523	0.1424	ns	0.4335	0.4658	ns
GM-CSF	0.9697	0.0063	**	0.217	0.7258	ns
CCL1	0.9978	0.0001	***	0.9223	0.0257	*
IL-1β	0.9259	0.024	*	0.4967	0.3947	ns
IL-2	0.997	0.0002	***	0.9622	0.0088	**
IL-4	0.839	0.0756	ns	0.3459	0.5685	ns
IL-6	0.9978	0.0001	***	0.2512	0.6836	ns
IL-10	0.7763	0.1226	ns	*Not included*	*Not included*	*Not included*
IL-16	0.9268	0.0235	*	-0.1057	0.8657	ns
IP-10	0.9396	0.0176	*	0.8975	0.0388	*
CXCL11	0.9991	<0.0001	****	0.3212	0.5982	ns
CXCL1	0.4165	0.4854	ns	-0.09931	0.8738	ns
CCL2	0.9962	0.0003	***	0.406	0.4977	ns
CCL7	0.9979	0.0001	***	0.2787	0.6498	ns
CCL22	0.9985	<0.0001	****	-0.1836	0.7675	ns
MIP-1α	0.5437	0.3435	ns	0.3033	0.6199	ns
MIP-1B	0.8665	0.0574	ns	0.06672	0.9151	ns
MIP-2	0.293	0.6324	ns	*Not included*	*Not included*	*Not included*
MIP-3α	0.45	0.447	ns	*Not included*	*Not included*	*Not included*
MIP-3β	0.5779	0.3075	ns	0.1752	0.7781	ns
CCL5	0.9977	0.0001	***	0.3167	0.6036	ns
CXCL16	0.9933	0.0007	***	0.1118	0.858	ns
CXCL12	0.7921	0.1102	ns	0.6467	0.2383	ns
CCL17	0.9827	0.0027	**	-0.1075	0.8634	ns
TNFα	0.9879	0.0016	**	0.1735	0.7802	ns

	BALB/c spleen IFNγ			CB6F1 spleen IFNγ		
Spleen cytokines	Pearson r	P (two tailed)	P value summary	Pearson r	P (two tailed)	P value summary
BCA-1 CXCL13	0.5481	0.3389	ns	-0.7482	0.1458	ns
CCL27	0.6234	0.2612	ns	0.1947	0.7537	ns
CXCL5	0.6349	0.2498	ns	-0.07908	0.8994	ns
CCL11	0.533	0.355	ns	-0.1941	0.7544	ns
CCL24	*Not included*	*Not included*	*Not included*	*Not included*	*Not included*	*Not included*
CX3CL1	0.6243	0.2603	ns	0.2123	0.7317	ns
GM-CSF	0.6268	0.2578	ns	0.9836	0.0025	**
CCL1	0.8179	0.0907	ns	0.9701	0.0062	**
IL-1β	0.706	0.1827	ns	0.7843	0.1163	ns
IL-2	0.8755	0.0518	ns	0.9008	0.037	*
IL-4	0.6419	0.243	ns	-0.3627	0.5485	ns
IL-6	0.894	0.0408	*	0.8129	0.0944	ns
IL-10	0.8075	0.0984	ns	0.2612	0.6713	ns
IL-16	-0.4137	0.4887	ns	-0.2392	0.6984	ns
IP-10	0.7396	0.1531	ns	0.7015	0.1868	ns
CXCL11	0.6019	0.2828	ns	0.3285	0.5894	ns
CXCL1	0.2744	0.6551	ns	-0.3434	0.5716	ns
CCL2	0.1704	0.7841	ns	0.6175	0.2671	ns
CCL7	0.317	0.6032	ns	0.05911	0.9248	ns
CCL22	0.6299	0.2547	ns	0.3301	0.5874	ns
MIP-1α	0.4771	0.4164	ns	-0.515	0.3745	ns
MIP-1β	0.5659	0.32	ns	0.6438	0.2411	ns
MIP-2	0.362	0.5493	ns	*Not included*	*Not included*	*Not included*
MIP-3α	*Not included*	*Not included*	*Not included*	*Not included*	*Not included*	*Not included*
MIP-3β	0.7098	0.1793	ns	0.02433	0.969	ns
CCL5	0.928	0.0229	*	0.8883	0.0441	*
CXCL16	0.642	0.2429	ns	-0.5451	0.342	ns
CXCL12	0.7075	0.1814	ns	-0.3522	0.561	ns
CCL17	0.6347	0.25	ns	-0.03762	0.9521	ns
TNFα	0.4839	0.4088	ns	0.4893	0.4028	ns

Supplementary Table 5: Correlations of BALB/c lungs (Log_{10} CFU/ml) and CB6F1 lungs (Log_{10} CFU/ml) mice vaccinated and challenged with H37Rv or HN878 versus other cytokines in the supernatants of M7 stimulated splenocytes and lung cells from mice vaccinated with BCG.

	BALB/c H37Rv lungs (Log_{10} CFU/ml)			CB6F1 H37Rv lungs (Log_{10} CFU/ml)		
**Lungs cytokines**	**Pearson r**	**P (two tailed)**	**P value summary**	**Pearson r**	**P (two tailed)**	**P value summary**
BCA-1 CXCL13	0.2366	0.7016	ns	-0.2617	0.6706	ns
CCL27	-0.3871	0.5197	ns	-0.306	0.6166	ns
CXCL5	-0.8169	0.0914	ns	0.001721	0.9978	ns
CCL11	-0.5839	0.3012	ns	-0.449	0.4481	ns
CCL24	0.3328	0.5843	ns	-0.519	0.3702	ns
CX3CL1	-0.6248	0.2598	ns	-0.5885	0.2966	ns
GM-CSF	-0.7513	0.1432	ns	0.03751	0.9523	ns
CCL1	-0.8684	0.0562	ns	-0.7915	0.1107	ns
IFNγ	-0.8416	0.0738	ns	-0.7663	0.1307	ns
IL-1β	-0.6915	0.1959	ns	-0.4033	0.5008	ns
IL-2	-0.8703	0.055	ns	-0.6612	0.2243	ns
IL-4	-0.5634	0.3227	ns	-0.4314	0.4682	ns
IL-6	-0.8268	0.0843	ns	0.1193	0.8485	ns
IL-10	-0.5325	0.3555	ns	*Not included*	*Not included*	*Not included*
IL-16	-0.6745	0.2117	ns	-0.415	0.4872	ns
IP-10	-0.6452	0.2397	ns	-0.59	0.295	ns
CXCL11	-0.8183	0.0904	ns	-0.3016	0.6219	ns
CXCL1	-0.331	0.5863	ns	0.2631	0.6689	ns
CCL2	-0.8271	0.084	ns	-0.2651	0.6664	ns
CCL7	-0.8468	0.0703	ns	-0.1381	0.8247	ns
CCL22	-0.8267	0.0843	ns	0.492	0.3999	ns
MIP-1α	-0.2656	0.6659	ns	-0.3879	0.5188	ns
MIP-1β	-0.7633	0.1332	ns	0.2825	0.6452	ns
MIP-2	-0.5001	0.3909	ns	*Not included*	*Not included*	*Not included*
MIP-3α	-0.4376	0.4612	ns	*Not included*	*Not included*	*Not included*
MIP-3β	-0.124	0.8425	ns	0.309	0.613	ns
CCL5	-0.8292	0.0825	ns	-0.03524	0.9551	ns
CXCL16	-0.8302	0.0818	ns	0.07186	0.9086	ns
CXCL12	-0.3728	0.5366	ns	-0.2304	0.7093	ns
CCL17	-0.7396	0.1531	ns	0.3222	0.597	ns
TNFα	-0.8345	0.0788	ns	0.1666	0.7889	ns

	BALB/c H37Rv lungs (Log_{10} CFU/ml)			CB6F1 H37Rv lungs (Log_{10} CFU/ml)		
**Spleen cytokines**	**Pearson r**	**P (two tailed)**	**P value summary**	**Pearson r**	**P (two tailed)**	**P value summary**
BCA-1 CXCL13	0.2366	0.7016	ns	0.9416	0.0168	*
CCL27	-0.3871	0.5197	ns	0.3089	0.613	ns
CXCL5	-0.8169	0.0914	ns	0.4506	0.4463	ns
CCL11	-0.5839	0.3012	ns	0.6827	0.204	ns
CCL24	0.3328	0.5843	ns	*Not included*	*Not included*	*Not included*
CX3CL1	-0.6248	0.2598	ns	0.1994	0.7478	ns
GM-CSF	-0.7513	0.1432	ns	-0.9169	0.0284	*
CCL1	-0.8684	0.0562	ns	-0.7331	0.1587	ns
IFNγ	-0.8416	0.0738	ns	-0.845	0.0716	ns
IL-1β	-0.6915	0.1959	ns	-0.6647	0.221	ns
IL-2	-0.8703	0.055	ns	-0.9109	0.0315	*
IL-4	-0.5634	0.3227	ns	0.8005	0.1037	ns
IL-6	-0.8268	0.0843	ns	-0.5992	0.2856	ns
IL-10	-0.5325	0.3555	ns	0.1562	0.802	ns
IL-16	-0.6745	0.2117	ns	0.4436	0.4543	ns
IP-10	-0.6452	0.2397	ns	-0.3183	0.6017	ns
CXCL11	-0.8183	0.0904	ns	0.07011	0.9108	ns
CXCL1	-0.331	0.5863	ns	0.6832	0.2036	ns
CCL2	-0.8271	0.084	ns	-0.481	0.4121	ns
CCL7	-0.8468	0.0703	ns	0.1411	0.8209	ns
CCL22	-0.8267	0.0843	ns	-0.03729	0.9525	ns
MIP-1α	-0.2656	0.6659	ns	0.6492	0.2359	ns
MIP-1β	-0.7633	0.1332	ns	-0.3241	0.5947	ns
MIP-2	-0.5001	0.3909	ns	*Not included*	*Not included*	*Not included*
MIP-3α	-0.4376	0.4612	ns	*Not included*	*Not included*	*Not included*
MIP-3β	-0.124	0.8425	ns	0.2894	0.6367	ns
CCL5	-0.8292	0.0825	ns	-0.5335	0.3545	ns
CXCL16	-0.8302	0.0818	ns	0.827	0.0841	ns
CXCL12	-0.3728	0.5366	ns	0.5509	0.3359	ns
CCL17	-0.7396	0.1531	ns	0.5254	0.3632	ns
TNFα	-0.8345	0.0788	ns	-0.07077	0.91	ns

	BALB/c HN878 lungs (Log_{10} CFU/ml)			CB6F1 HN878 lungs (Log_{10} CFU/ml)		
**Lungs cytokines**	**Pearson r**	**P (two tailed)**	**P value summary**	**Pearson r**	**P (two tailed)**	**P value summary**
BCA-1 CXCL13	0.09206	0.883	ns	-0.001461	0.9981	ns
CCL27	-0.5101	0.3799	ns	-0.2358	0.7026	ns
CXCL5	-0.7068	0.1819	ns	-0.1351	0.8285	ns
CCL11	-0.5879	0.2971	ns	-0.5687	0.3171	ns
CCL24	0.4419	0.4563	ns	-0.2913	0.6344	ns
CX3CL1	-0.6838	0.203	ns	-0.5319	0.3562	ns
GM-CSF	-0.6504	0.2347	ns	-0.2004	0.7466	ns
CCL1	-0.7857	0.1152	ns	-0.7967	0.1066	ns
IFNγ	-0.7467	0.1471	ns	-0.8938	0.0409	*
IL-1β	-0.6501	0.235	ns	-0.2652	0.6663	ns
IL-2	-0.7891	0.1125	ns	-0.8605	0.0612	ns
IL-4	-0.5636	0.3225	ns	-0.2989	0.6252	ns
IL-6	-0.7252	0.1656	ns	-0.09111	0.8842	ns
IL-10	-0.5681	0.3177	ns	*Not included*	*Not included*	*Not included*
IL-16	-0.5774	0.308	ns	-0.1191	0.8487	ns
IP-10	-0.5644	0.3216	ns	-0.7515	0.143	ns
CXCL11	-0.7194	0.1708	ns	-0.2843	0.6429	ns
CXCL1	-0.4491	0.448	ns	0.1966	0.7513	ns
CCL2	-0.7124	0.1769	ns	-0.4068	0.4967	ns
CCL7	-0.7637	0.1329	ns	-0.1372	0.8259	ns
CCL22	-0.7216	0.1688	ns	0.3153	0.6054	ns
MIP-1α	-0.3363	0.58	ns	-0.1743	0.7793	ns
MIP-1β	-0.7723	0.1258	ns	0.06614	0.9158	ns
MIP-2	-0.6897	0.1976	ns	*Not included*	*Not included*	*Not included*
MIP-3α	-0.5295	0.3588	ns	*Not included*	*Not included*	*Not included*
MIP-3β	-0.07988	0.8984	ns	0.2447	0.6916	ns
CCL5	-0.7422	0.1509	ns	-0.186	0.7646	ns
CXCL16	-0.7532	0.1416	ns	-0.07952	0.8989	ns
CXCL12	-0.3186	0.6014	ns	-0.3951	0.5103	ns
CCL17	-0.636	0.2488	ns	0.2473	0.6883	ns
TNFα	-0.7548	0.1402	ns	-0.08213	0.8955	ns

	BALB/c HN878 lungs (Log_{10} CFU/ml)			CB6F1 HN878 lungs (Log_{10} CFU/ml)		
**Spleen cytokines**	**Pearson r**	**P (two tailed)**	**P value summary**	**Pearson r**	**P (two tailed)**	**P value summary**
BCA-1 CXCL13	0.09206	0.883	ns	0.9324	0.0209	*
CCL27	-0.5101	0.3799	ns	0.3507	0.5628	ns
CXCL5	-0.7068	0.1819	ns	0.5593	0.327	ns
CCL11	-0.5879	0.2971	ns	0.6263	0.2583	ns
CCL24	0.4419	0.4563	ns	*Not included*	*Not included*	*Not included*
CX3CL1	-0.6838	0.203	ns	-0.02558	0.9674	ns
GM-CSF	-0.6504	0.2347	ns	-0.8841	0.0466	*
CCL1	-0.7857	0.1152	ns	-0.6735	0.2127	ns
IFNγ	-0.7467	0.1471	ns	-0.8313	0.081	ns
IL-1β	-0.6501	0.235	ns	-0.867	0.0571	ns
IL-2	-0.7891	0.1125	ns	-0.9807	0.0032	**
IL-4	-0.5636	0.3225	ns	0.7431	0.1501	ns
IL-6	-0.7252	0.1656	ns	-0.4683	0.4263	ns
IL-10	-0.5681	0.3177	ns	0.168	0.7871	ns
IL-16	-0.5774	0.308	ns	0.1017	0.8707	ns
IP-10	-0.5644	0.3216	ns	-0.1921	0.7569	ns
CXCL11	-0.7194	0.1708	ns	0.1689	0.786	ns
CXCL1	-0.4491	0.448	ns	0.6446	0.2403	ns
CCL2	-0.7124	0.1769	ns	-0.4185	0.4831	ns
CCL7	-0.7637	0.1329	ns	0.3043	0.6187	ns
CCL22	-0.7216	0.1688	ns	0.1307	0.8341	ns
MIP-1α	-0.3363	0.58	ns	0.7617	0.1345	ns
MIP-1β	-0.7723	0.1258	ns	-0.2162	0.7269	ns
MIP-2	-0.6897	0.1976	ns	*Not included*	*Not included*	*Not included*
MIP-3α	-0.5295	0.3588	ns	*Not included*	*Not included*	*Not included*
MIP-3β	-0.07988	0.8984	ns	0.3322	0.585	ns
CCL5	-0.7422	0.1509	ns	-0.5887	0.2963	ns
CXCL16	-0.7532	0.1416	ns	0.6328	0.2519	ns
CXCL12	-0.3186	0.6014	ns	0.6637	0.2219	ns
CCL17	-0.636	0.2488	ns	0.5544	0.3322	ns
TNFα	-0.7548	0.1402	ns	-0.3301	0.5874	ns

Supplementary Table 6: Correlations of BALB/c lungs (Log_{10} CFU/ml) and CB6F1 lungs (Log_{10} CFU/ml) mice vaccinated and challenged with H37Rv or HN878 versus IFN-γ secreting cells evaluated by ex vivo ELISPOT (SFC/million).

	Spleen IFNγ (SFC/million)			Lungs IFNγ (SFC/million)		
	**Pearson r**	**P (two tailed)**	**P value summary**	**Pearson r**	**P (two tailed)**	**P value summary**
**BALB/c H37Rv lungs (Log_{10} CFU/ml)**	-0.7299	0.1615	ns	-0.9253	0.0242	*
**CB6F1 H37Rv lungs (Log_{10} CFU/ml)**	-0.5692	0.3166	ns	-0.7291	0.1622	ns
**BALB/c HN878 lungs (Log_{10} CFU/ml)**	-0.8078	0.0982	ns	-0.8729	0.0533	ns
**CB6F1 HN878 lungs (Log_{10} CFU/ml)**	-0.2985	0.6257	ns	-0.8625	0.0599	ns
